# Isolation and antimicrobial susceptibility pattern of *Staphylococcus aureus* in patients with surgical site infection at Debre Markos Referral Hospital, Amhara Region, Ethiopia

**DOI:** 10.1186/2049-3258-72-16

**Published:** 2014-05-28

**Authors:** Amlsha Kahsay, Adane Mihret, Tamrat Abebe, Tebkew Andualem

**Affiliations:** 1 Department of Microbiology, Immunology and Parasitology, School of Medicine, Addis Ababa University, Addis Ababa, Ethiopia; 2 School of Medicine, College of Medicine and Health Sciences, Debre Markos University, Debre Markos, Ethiopia; 3 Armauer Hansen Research Institute, Addis Ababa, Ethiopia; 4 Department of Biochemistry, WHO Immunology Research and Training Center, University of Lausanne, Lausanne, Switzerland; 5 Debre Markos Referral Hospital, Debre Markos, Ethiopia

**Keywords:** *S. aureus*, MRSA, Surgical site infection, Prevalence, Antibiotic susceptibility

## Abstract

**Background:**

*Staphylococcus aureus,* especially Methicillin Resistant Staphylococcus Aureus (MRSA) is a major health problem recognized as the most important nosocomial pathogen, often causing postoperative wound infections. Antibiotic resistance by MRSA has grown to be common, and resistance to almost all antibiotics has been found among these strains. The aim of this study was to determine the prevalence, antimicrobial susceptibility patterns and associated risk factors of *S. aureus* in patients with surgical site infections in an Ethiopian hospital.

**Methods:**

A cross-sectional study was conducted from December 1, 2011 to March 30, 2012 among patients with surgical site infections at Debre Markos Referral Hospital, Debre Markos, Ethiopia. All wound swabs obtained from patients with surgical site infections during the study period were cultured on mannitol salt agar media which is selective for *S. aureus*. Isolated strains of *S. aureus* were tested for antibiotic susceptibility patterns using standard disc diffusion technique, and interpretation of resistance was done based on Clinical and Laboratory Standard Institute criteria. Univariate and multivariable analyses were used to assess the risk factors.

**Results:**

Of the 184 surgical patients who had developed surgical site infection, *S. aureus* was isolated from 73 (39.7%) cases. Out of the 73 isolates of *S. aureus,* 36 (49.7%) were MRSA. Among the study participants, prevalence of MRSA was found to be 19.6%. The clinical isolates showed >80% level of resistance to ampicillin, amoxicillin, penicillin G, erythromycin, gentamicin and cotrimoxazole whereas <50% level of resistance was observed against clindamycin, oxacillin, tetracycline and vancomycin. MRSA strains showed resistance ranging from 5.6% (vancomycin) to 100% (cotrimoxazole). Of the following risk factors: sex, age, pus consistency, duration of operation, type of surgery, ward and hospital stay, laparotomy type of surgery was identified as a risk factor for infection by *S. aureus*.

**Conclusion:**

The prevalence of *S. aureus* and/or MRSA infection in surgical and gynaecology & obstetrics wards of Debre Markos Referral Hospital was found to be high. The majority of isolates were highly resistant to major antimicrobial agents.

## Background

*S. aureus* has been known as a cause of deep-seated wound infection for close to a century, having been recognized as a cause of nosocomial infection and super-infection in patients receiving antimicrobial agents such as surgical cases [1]. MRSA colonizing the anterior nares and skin of humans are the major sources of surgical site infection as well as nosocomial spread. Several trends have been identified in the epidemiology of MRSA infections: i) increasing incidence of MRSA infections, particularly among surgical patients [2]; ii) increasing proportion of nosocomial infections caused by MRSA. By the early 1990s, MRSA accounted for 20% - 25% of *S. aureus* isolates from hospitalized patients. In 1999, MRSA accounted for >50% of *S. aureus* isolates from patients in ICUs in the National Nosocomial Infection Surveillance (NNIS) system. In 2003, 59.5% of *S. aureus* isolates in NNIS ICUs were MRSA [3,4]; and iii) increasing level of resistance to commonly prescribed antibiotics [5]. Various risk factors including excessive use of antibiotics and prolonged hospitalization have been identified, which are associated with promotion of colonization and infection to surgical wounds as well as spread of MRSA in the health care settings [6,7].

Data are scarce in resource-limited countries. Furthermore, isolation and antibiogram profiles require periodical monitoring, especially in settings like Ethiopia, where surgical site infection accounts for considerable morbidity and mortality rates [8]. Unfortunately, there were no local guidelines for prophylaxis and treatment of *S. aureus* infection at the study area. In the study area, there had been no previous study related to this topic. Hence, the present study was aimed at determining the prevalence and antimicrobial resistance pattern of *S. aureus* among patients who developed surgical site infection.

Therefore, this study may help to understand the contribution of *S. aureus* and MRSA for the development of surgical site infection. This study can potentially identify the high-risk groups of the study population for surgical site infection. Moreover, it could provide useful information that can optimize the potential benefits of isolation and antimicrobial susceptibility testing in *S. aureus* and MRSA control. It can also provide baseline information for future research in this area.

## Methods

### Study area

The study was conducted at Debre Markos Referral Hospital, Amhara Region, Ethiopia, in surgical and gynaecology and obstetrics wards. Debre Markos Town is located 300 kms North-West of Addis Ababa. Debre Markos Referral Hospital is one of the hospitals governed by Amhara Regional Health Bureau, Ethiopia, and it serves about 3.5 million people.

### Swab sample and study population

This study was conducted from December 1, 2011 to March 30, 2012. Swab samples were collected from admitted patients suspected of surgical site infections.

### Inclusion criteria

Our inclusion criteria was having surgical wound with pus discharge, serous or seropurulent discharge, signs of sepsis (warmth, erythema, induration and pain) and with physician diagnosis suspected of surgical site infection.

Suspected of surgical site infection mean having the above signs and symptoms assessed by senior physicians (surgeon and gynecologist) based on CDC criteria from surgical and gynecology wards (there is no orthopedic ward in the hospital).

### Sample collection and handling

Following guidelines of Clinical and Laboratory Standard Institute (CLSI), from surgical patients developing postoperative wound infections, swab samples were collected from the depths of the wound using a sterile cotton swab under an aseptic condition. Then specimens were transferred into a sterile nutrient broth in a screw caped test tube and were delivered to the laboratory within 1 hour.

### Culture and identification

The wound swabs were inoculated directly on mannitol salt agar (Oxoid) and sub cultured to blood agar (Oxoid). All positive cultures were identified by their characteristic appearance on media, gram staining reactions and the pattern of biochemical profiles using standard procedures. *S. aureus* was identified based on characteristic yellow colony surrounded by yellow zone on mannitol salt agar; β hemolytic colonies with yellowish pigment on blood agar; gram positive cocci singly in pair, in short chain or clusters, catalase positive and coagulase production and mannitol fermentation.

### Antimicrobial susceptibility testing

The antimicrobial susceptibility testing of all *S. aureus* isolates were done according to the criteria of CLSI by disc diffusion method [9].

From a pure culture, 3–5 selected colonies of *S. aureus* had been taken and transferred to a tube containing 5 ml sterile nutrient broth (Oxoid) and were mixed gently. Then a homogenous suspension was formed and incubated at 37°C until the turbidity of the suspension became adjusted to a 0.5 McFarland Standard (Bacterial concentration of 1.5 × 10^8^ colony forming unit/ml).

A sterile cotton swab was used and the excess suspension was removed by gentle rotation of the swab against the surface of the tube. The swab was then used to distribute the bacteria evenly over the entire surface of Mueller Hinton plate (pH 7.2-7.4) (Oxoid).

The inoculated plates were left at room temperature to dry for 3–5 minutes and a set of 10 antibiotic discs (Oxoid) with the following concentrations were then evenly distributed on the surface of a Muller Hinton plate: amoxicillin (AML) (30 μg), ampicillin (AMP) (10 μg), penicillin G (P) (10 mu g), erythromycin (E) (15 μg), gentamicin (CN) (10 μg), oxacillin (Ox) (1 μg), clindamycin (DA) (2 μg), tetracycline (TE) (30 μg), cotrimoxazole (SXT) (25 μg) and vancomycin (VA) (30 μg). The criteria used to select the antimicrobial agents to be tested were based on their availability and frequent prescriptions for the management of wound infections in the hospital.

The plates were then incubated at 37°C for 24 hours. Diameters of zones of inhibition around the discs were measured to the nearest millimeter using a metal caliper, and the isolates were classified as sensitive, intermediate and resistant according to the standardized table supplied by the CLSI. *S. aureus* ATCC 25923 was used as a quality control for culture and antimicrobial susceptibility testing throughout the study.

### Definitions

#### MRSA

A type of staphylococcus bacteria that is resistant to certain antibiotics called beta-lactams. These antibiotics include methicillin and other more common antibiotics such as oxacillin, penicillin, and amoxicillin.

#### Surgical site infection

An infection that occurs after surgery in the part of the body where the surgery took place.

#### Induration

A focus or region of abnormally hardened tissue.

### Statistical analysis

All data were analyzed using Statistical Package for Social Sciences (SPSS) version 16. Bivariate and multivariable logistic regression model was used to ascertain the association between the different variables and the outcome variable with respective wadan-test to identify the possible risk factors. All statistical tests were two-tailed, and values of p < 0.05 were considered as statistically significant.

### Ethical clearance

The protocol was approved by institution of review board of College of Health Sciences, Addis Ababa University. To conduct this study, a letter of permission was also obtained from the administration of Debre Markos Referral Hospital. Written informed consent was obtained from each patient. During the study period, all findings were reported to the attending physicians.

## Results

### Socio-demographic characteristics of study participants

In the present study, a total of 184 adult study participants were included: 67 from the surgical and 117 from gynaecology & obstetrics wards. In this study, 61 (33.2%) males and 123 (68.8%) females were participated. The mean age was 35 years with median and standard deviation of 5 and 14.4 respectively (see Table 1).

**Table 1:** Socio-demographic characteristics of patients with surgical site infection at Debre Markos Referral Hospital, Amhara, Ethiopia [Dec. 2011-March 2012]

**Variables**	**Frequency**	**%**
**Age**		
18-30	100	54.3
31-40	43	23.4
41-50	18	9.8
≥51	23	12.5
**Sex**		
Male	61	33.2
Female	123	68.8
**Education**		
Elementary & junior	39	21.2
Secondary & above	49	26.6
Illiterate	96	52.2
**Residence**		
Urban	65	35.3
Rural	119	64.7
**Occupation**		
Governmental employee	23	12.5
Farmer	97	52.7
Student	6	3.3
House wife	53	28.8
Other	5	2.7

### Patterns of admission

Of the 67 patients from surgical ward, procedures related to gastrointestinal problems accounted for 74.6% (50 patients), followed by gangrene of different tissues 9.0% (6 patients), genitourinary tract problems 7.5% (5 patients), lymphoma 4.5% (3 patients), hernia accounted for 3.0% (2 patients) and the remaining 1.5% (1 patient) was germ cell tumor. Of the 117 obstetrics and gynecological cases, cesarean section accounted for 82.9% (97 patients), followed by cervical cancer 5.1% (6 patients), ovarian cancer 4.3% (5 patients), myoma accounted for 3.4% (4 patients) and other gynecological problems 4.3% (5 patients) of the cases.

### Duration of hospital stay

Among the study participants, most (62%) stayed for ≤ 5 days with a mean and standard deviation of 6.1 and 3.9 respectively. The length of stay in the hospital varied from 3 to 24 days.

### Prevalence of S. aureus and MRSA

Of 184 patients, 73 (39.7%) developed *S. aureus* infections based on the clinical evaluations and positive wound culture results (see Table 2). Of the study participants, 21(28.8%) were males and 52 (71.2%) were females. Out of the 73 patients 21 (31.3%) were from surgical wards and 52 (44.4%) were from gynaecology and obstetrical wards.

**Table 2:** **Prevalence of *S. aureus *** **and MRSA in different age groups among patients with surgical site infection at Debre Markos Referral Hospital, Amhara, Ethiopia [December, 2011 – March, 2012]**

**Age in years**	**Surgical cases (n = 67)**		**Gynaecology cases (n = 117)**		**Total cases (n = 184)**	
	***S. aureus***	**MRSA**	***S. aureus***	**MRSA**	***S. aureus***	**MRSA**
	**no. (%)**	**no. (%)**	**no. (%)**	**no. (%)**	**no. (%)**	**no. (%)**
18-30	5 (7.5)	2 (2.9)	32 (27.4)	18 (15.4)	37 (20.1)	20 (10.9)
31-40	4 (5.9)	1 (1.5)	15 (12.8)	6 (5.1)	19 (10.3)	7 (3.8)
41-50	3 (4.5)	2 (2.9)	4 (3.4)	3 (2.6)	7 (3.8)	5 (2.7)
≥51	9 (13.4)	3 (4.5)	1 (0.8)	1(0.8)	10 (5.4)	4 (2.2)
**Total**	**21 (31.3)**	**8 (11.9)**	**52 (44.4)**	**28 (23.9)**	**73 (39.7)**	**36 (19.6)**

**Figure 1:**
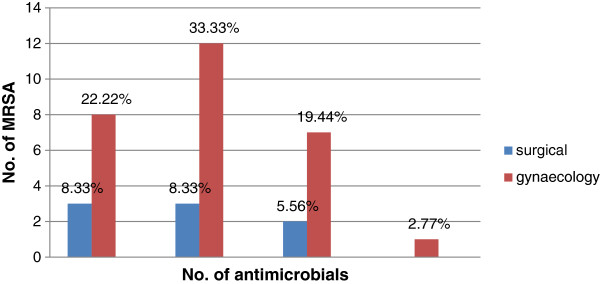
MRSA strains showing resistance for 6 or more antimicrobial agents tested at Debre Markos Referral Hospital, Amhara, Ethiopia [December, 2011 - March, 2012].

Out of the 184 patients, 36 (19.6%) developed MRSA infection; 28 (77.8%) of them were from gynecology and obstetrical wards and the rest 8 (22.2%) were from surgical wards (see Table 2). MRSA strains accounted for 38.1% and 53.8% isolates from surgical patients and gynaecology and obstetrics cases respectively. The resistance pattern of MRSA isolates to different antimicrobials is shown in Figure 1.

### Risk factors associated with development of S. aureus infection

Risk factors associated with development of *S. aureus* infection were analysed using bivariate analysis with the following findings. A statistically significant association was noted with laparotomy type of surgery (OR = 3.92, 95% CI = 1.82-8.43, p -value = 0.0001), clinical symptom of induration (OR = 0.53, 95% CI = 0.28-0.99, p -value = 0.049) and duration of operation ≥61 minutes (OR = 2.93, 95% CI = 1.46-5.91, p -value = 0.003).

However, in multivariable logistic regression analysis, laparotomy type of surgery showed a significant association with *S. aureus* infection. As shown in Table 3, patients who had undergone laparotomy type of surgery were 2.03 times more likely to develop infection with *S. aureus* (OR = 2.03, 95% CI = 1.91-7.01) than other types of surgery.

**Table 3:** **Bivariate and multivariable analysis of characteristics related with *S. aureus *** **isolation at Debre Markos Referral Hospital, Amhara, Ethiopia [December, 2011 - March, 2012]**

**Variables**	**Total**	***S. aureus *** **positive**	***S. aureus *** **negative**	**COR (95% CI)**	**p-value**	**AOR (95% CI)**	**p-value**
**Age**							
18-30	100	37	63	1			
31-40	43	19	24	0.74 (0.35-1.53)	0.420		
41-50	18	7	11	0.92 (0.32-2.58)	0.879		
≥51	23	10	13	0.76 (0.30-1.91)	0.565		
**Sex**							
Female	123	52	71	1			
Male	61	21	40	1.39 (0.73-2.64)	0.306		
**Pus consistency**							
Yellowish	126	58	68	1			
Bloody	49	15	34	1.93 (0.95-3.89)	0.065		
**Duration of operation**							
≤30 minute	92	46	46	1		1	
31-60 minute	29	11	18	1.63 (0.69-3.84)	0.258	1.40 (0.41-4.14)	0.29
≥61 minute	63	16	47	2.93 (1.46-5.91)	0.003	0.34 (0.73-2.13)	0.49
**Type of surgery**							
Caesarean section	86	44	42	1		1	
Laparotomy	57	12	45	3.92 (1.82-8.43)	0.0001	2.03 (1.91-7.01)	0.03
Appendectomy	12	5	7	1.46 (0.43-4.98)	0.539	0.78 (0.08-7.65)	0.83
Hysterectomy	9	4	5	1.31 (0.32-5.21)	0.702	0.56 (0.05-5.74)	0.62
Prostectomy	4	2	2	1.04 (0.14-7.78)	0.964	0.01 (0.91-8.01)	0.99
Salphangectomy	3	1	2	2.09 (0.18-3.97)	0.552	1.40 (0.06-17.98)	0.95
Others	13	5	8	1.67 (0.50-5.53)	0.397	0.03 (0.11-11.30)	0.99
**Induration**							
No	69	21	48	1		1	
Yes	115	52	63	0.53 (0.28-0.99)	0.049	0.60 (0.31-1.17)	0.14
**Ward**							
Surgical	67	21	46	1			
Gynaecology	117	52	65	0.57 (0.30-1.07)	0.082		
**Hospital stay**							
≤5 days	114	49	65	1			
6-10 days	55	18	37	1.55 (0.78-3.04)	0.203		
11-15 days	5	1	4	3.01 (0.32-27.83)	0.330		
≥16 days	10	5	5	0.75 (0.20-2.74)	0.669		

1= reference category induration- a focus or region of abnormally hardened tissue.

### Antimicrobial susceptibility pattern

The antimicrobial susceptibility patterns of 73 *S. aureus* isolates was determined against 10 antimicrobial agents as presented in Table 4. The majority (>80%) of the isolates were resistant to the following antibiotics: ampicillin, amoxicillin, penicillin G, erythromycin, gentamicin and cotrimoxazole. *S. aureus* isolates showed <50% of resistance against vancomycin, oxacillin, tetracycline and clindamycin.

**Table 4:** **Antimicrobial susceptibility pattern of *S. aureus *** **and MRSA from patients with surgical site infection at Debre Markos Referral Hospital, Amhara, Ethiopia [December, 2011 - March, 2012]**

	***S. aureus *** **(n = 73)**			**MRSA (n = 36)**		
**Antimicrobial agent**	**Sensitive no. (%)**	**Intermediate no. (%)**	**Resistant no. (%)**	**Sensitive no. (%)**	**Intermediate no. (%)**	**Resistant no (%)**
Ampicillin^a^	13 (17.8)	-	60 (82.2)	-	-	36 (100)
Amoxicillin^a^	13 (17.8)	-	60 (82.2)	-	-	36 (100)
Erythromycin^a^	-	3 (4.1)	70 (95.9)	-	1 (2.8)	35 (97.2)
Gentamicin^a^	9 (12.3)	-	64 (87.7)	2 (5.6)	-	34 (94.4)
Tetracycline^a^	37 (50.7)	12 (16.4)	24 (32.9)	12 (33.3)	9 (25)	15 (41.7)
Penicillin G^a^	13 (17.8)	-	60 (82.2)	-	-	36 (100)
Clindamycin^a^	7 (9.6)	31 (42.4)	35 (47.9)	2 (5.6)	12 (33.3)	22 (61.1)
Cotrimoxazole^a^	1 (1.4)	1 (1.4)	71 (97.2)	-	-	36 (100)
Vancomycin^a^	70 (95.9)	-	3 (4.1)	34 (94.4)	-	2 (5.6)
Oxacillin^a^	37 (50.7)	-	36 (49.3)	-	-	36 (100)

^a^Clinical and Laboratory Standards Institute (CLSI) breakpoint for Staphylococcus spp.

AMP: ampicillin, AML: amoxicillin, P: penicillin G, CN: gentamicin, E: erythromycin, Ox: oxacillin, TE: tetracycline, DA: clindamycin, SXT: cotrimoxazole, VA: vancomycin.

Intermediate resistant: The “intermediate” category includes isolates with antimicrobial MICs that approach usually attainable blood and tissue levels and for which response rates may be lower than for susceptible isolates (CLSI definition).

However, MRSA strains showed resistance ranging from 5.6% (vancomycin) to 100% (ampicillin, amoxicillin, penicillin G and cotrimoxazole). More than 40% of the MRSA isolates showed multiple resistance (except vancomycin), and 33.3% of these isolates showed intermediate susceptibility to clindamycin (see Table 4).

Of the 73 isolates tested for antimicrobial susceptibility, 36 (49.3%) were found to show resistance to oxacillin and hence defined as MRSA isolates. MRSA isolates from surgical patients as well as gynaecology and obstetrics cases showed multiple resistances (≥6 antimicrobial).

## Discussion

The development and treatment of surgical wound infections have always been limiting factors to the success of surgical treatment. Although continuous improvements have been made, surgical site infections continue to occur at an unacceptable rate, annually costing billions of dollars in economic loss caused by associated morbidity and mortality [8]. *S. aureus* has long been recognized as an important pathogen in human disease and is the most common cause of nosocomial infections [1].

A study conducted on a murine model showed that laparotomy type of surgery had a statistically significant association with *S. aureus* infection [10]. Consistent with this, the present finding also showed that the odds of favoring *S. aureus* infection in cases undergone laparotomy type of surgery was 2.03 times more than other types of surgery (see Table 3). This could be explained by this type of surgery which had open wound and can easily be contaminated by this bacterium.

Greater than 80% of resistance was observed to ampicillin, amoxicillin, penicillin G, gentamicin, erythromycin and cotrimoxazole among *S. aureus* isolates (see Table 4). Many factors may have contributed to such level of resistance, including misuse of antibiotics by health professionals, unskilled practitioners and lay persons. In Debre Markos it is a common practice that antibiotics can be purchased without prescription, which leads to misuse of antibiotics by the public, thus contributing to the emergence and spread of antimicrobial resistance. Other causal factors could be poor hospital hygienic conditions, accounting for the spread of resistant bacteria and inadequate surveillance, i.e. lack of information from routine antimicrobial susceptibility testing of bacterial isolates and surveillance testing of bacterial isolates and surveillance of antibiotic resistance, all of which are crucial for good clinical practice and for rational policies against antibiotic resistance [4]. Whereas <50% of resistance was observed by *S. aureus* isolates against vancomycin, oxacillin, tetracycline and clindamycin from all wards, in agreement with previous reports in Ethiopia and India [11,12].

All MRSA isolates encountered in this study were completely resistant to antibiotics, such as cotrimoxazole and erythromycin. A similar result was noted for erythromycin among MRSA strains from Trinidad and New York [13,14]. Similarly a comparable result was reported for cotrimoxazole in Islamabad [15].

The resistance rate of MRSA isolates to vancomycin was found to be 5.6% (see Table 4). This result was higher when compared to previous findings from different parts of the world, including Ethiopia. These reports revealed that there is no MRSA isolate resistance to vancomycin [11,15,16]. This variation may be due to timely emergence of resistance strains. However, our result is lower in contrast to report from New York (10%) [2]. This lower finding may be explained by method variation, since assessment of the previous study was in a controlled clinical trial.

In this study, high prevalence of multidrug resistant MRSA was observed. This may predispose patients to infection with intractable isolates and emphasizing the need for improved infection control practices and guidelines for use of antibiotics in this setting. Moreover, all MRSA strains isolated in our investigation were resistant to ≥3 antibiotics tested excluding β lactams (penicillin G, ampicillin, amoxicillin). Arora *et al*. revealed that, 73% of MRSA strains were multidrug resistant [17]. This indicated that resistant strains were emerged and the emergence of those resistant strains, especially for the most bactericidal anti-MRSA agents, may have further aggravated the emergence of multidrug resistant MRSA, and it may threaten the success of an MRSA control program. Molecular confirmation of MRSA strains was not conducted due to absence of molecular techniques in the study set up.

## Conclusions

The prevalence of *S. aureus* and MRSA in surgical and gynaecology and obstetrics wards of Debre Markos Referral Hospital were found to be high. The rate of MRSA strains among clinical isolates was also high. Vancomycin was relatively effective drug for *S. aureus* and MRSA infections. High level of resistance was observed to erythromycin, cotrimoxazole and gentamycin among *S. aureus* isolates from surgical ward. In addition, high level of resistance was observed to erythromycin, cotrimoxazole, gentamycin, ampicillin, amoxicillin and penicillin G among isolates from gynaecology and obstetrics ward. Moreover, all MRSA isolates were multidrug resistant (≥6). As a recommendation, the rationale of some antibiotic combinations requires evaluation and the establishment of antibiotic policy and treatment guidelines.

## Abbreviations

MRSA: Methicillin resistant *Staphylococcus aureus*; CLSI: Clinical Laboratory and Standards Institute; NNIS: National nosocomial infection surveillance.

## Competing interests

The authors’ declared that there was no personal and organizational conflicts during the thesis work.

## Authors’ contributions

AK: conception and initiation of the study, design, implementation, analysis and writing of the thesis work (first-author). TA: design, implementation, guidance, comments and co-writing of the thesis work. AM: design, implementation, comments and co-writing of the thesis work. TA: guidance, comments and unreserved support in facilitating good working environment. All authors read and approved the final manuscript.

## Authors’ information

Amlsha Kahsay (BSc, MSc), Lecturer in the School of Medicine, College of Health Sciences, Debre Markos University, Ethiopia. Tamrat Abebe (BSc, MSc, PhD candidate), Lecturer in the Department of Microbiology, Immunology and Parasitology, School of Medicine, Addis Ababa University, Ethiopia. Adane Mihret (DVM, MSc, PhD candidate): Lecturer in the Department of Microbiology, Immunology and Parasitology, School of Medicine, Addis Ababa University, Ethiopia. Tebkew Andualem (MD, Surgeon): Surgical Specialist at Debre Markos Referral Hospital, Ethiopia.
